# A Cross-Sectional Study on the Affordable Care Act from the Perspective of People Living with HIV: The Interplay between Knowledge, Stigma, Trust, and Attitudes

**DOI:** 10.1155/2020/6081721

**Published:** 2020-12-08

**Authors:** Christopher Kaperak, Sarah Elwood, Tamara Saint-Surin, Christopher Winstead-Derlega, Robert O. Brennan, Rebecca Dillingham, Kathleen A. McManus

**Affiliations:** ^1^School of Medicine, University of Virginia, Charlottesville 22908, VA, USA; ^2^Division of Infectious Diseases and International Health, University of Virginia, Charlottesville 22908, VA, USA; ^3^Infectious Diseases Associates of Central Virginia, Lynchburg 24501, VA, USA; ^4^Center for Health Policy, University of Virginia, Charlottesville 22908, VA, USA

## Abstract

**Background:**

Many AIDS Drug Assistance Programs (ADAPs) purchased Affordable Care Act (ACA) Qualified Health Plans (QHPs) for low-income people living with HIV (PLWH). To date, little has been published about PLWH's perspective on the ACA. We explored ACA knowledge, HIV stigma, trust in the healthcare system, and ACA attitudes among PLWH with ADAP-funded QHPs in Virginia.

**Methods:**

Participants were surveyed about demographic characteristics, ACA knowledge, HIV stigma, trust in various healthcare and government entities, and attitudes toward the ACA. Descriptive statistics were used. We assessed for associations (1) between baseline characteristics and correct ACA knowledge, HIV-related stigma, trust, and ACA attitudes and (2) between correct ACA knowledge and the following data: sources of ACA knowledge, HIV stigma, and trust.

**Results:**

Participants (*n* = 53) were a vulnerable population based on the assessment of social determinants of health, and 30% had correct ACA knowledge. Almost three-fourths of participants used HIV clinic case managers for ACA information. Participants who used websites for ACA information had correct ACA knowledge more often compared to those that did not (71% vs. 15%; *p* = 0.001). Those with correct ACA knowledge had lower stigma scores compared to those without correct ACA knowledge (93.8; SD: 15.4 vs. 108; SD: 20.3; *p* = 0.01). Participants trusted HIV clinicians more than general clinicians and insurance companies. No association was found between having correct ACA knowledge and endorsing having enough information about the ACA to understand how it will impact their HIV care.

**Conclusions:**

Websites imparted accurate ACA information. HIV clinic case managers were the most used source, and HIV clinicians were a trusted source of information. HIV clinicians and case managers should consider disseminating information about the ACA and its impact on HIV care delivery via internet videos. Lack of internet and stigma are a threat to PLWH gaining actionable healthcare information.

## 1. Introduction

With the implementation of the Affordable Care Act (ACA), HIV healthcare delivery and health insurance coverage for many people living with HIV (PLWH) in the United States (US) changed [1]. Across the US, many PLWH with low incomes gained insurance coverage through expanded Medicaid [1]. Additionally, even for those who did not receive Medicaid due to income restrictions or living in a Medicaid nonexpansion state, many aspects of HIV care changed [2]. Almost all state AIDS Drug Assistance Programs (ADAPs) offered to purchase ACA Qualified Health Plans (QHPs) for PLWH with low incomes [2, 3]. Virginia ADAP paid the insurance premiums, deductibles, and medication copayments, so most of the possible financial costs related to coverage through the ACA were covered by the state [2]. Our group and others have published studies demonstrating that PLWH with ADAP-funded QHPs are more likely to achieve viral suppression compared to PLWH who receive medications directly from a state ADAP [4–6].

In addition to the available quantitative data suggesting the importance of comprehensive insurance coverage for PLWH, the Kaiser Family Foundation has performed two focus groups with PLWH in urban centers to understand their experience with the ACA [7, 8]. These studies' participants with QHP coverage reported putting a lot of trust in case managers to help them make QHP enrollment decisions and prioritizing being able to continue to see their established HIV clinician [7, 8]. Our group also published a qualitative study about the perspectives and opinions of nonurban PLWH in Virginia who enrolled in ADAP-funded QHPs [9].

Besides the previously mentioned work, little else has been published about the ACA from the perspective of PLWH. We aimed to add more from the patient perspective. Additionally, with this descriptive, hypothesis-generating study, our group sought to understand the interplay of ACA knowledge, stigma, trust, and attitudes about the ACA, which have not been examined together, to our knowledge. In terms of knowledge, previous studies had demonstrated knowledge gaps about the ACA for PLWH in Nebraska [10]. We examined this topic in a nonurban southern population and added the examination of associations between ACA knowledge and stigma, trust, and attitudes about the ACA. These three patient-centered topics were chosen based on the review of previous research. We hypothesized that these could influence PLWH's decisions about healthcare and might be important areas to consider in thinking about how to convey information about changes in healthcare delivery to PLWH more effectively. For stigma, it has been identified as one of the most significant barriers to ending the HIV epidemic worldwide [11] and has been shown to mediate the relationship between self-efficacy and HIV medication adherence and quality of life [12]. We wondered if stigma may be associated with less self-efficacy in making decisions about the ACA. For trust, the Kaiser Family Foundation's work in urban focus groups has highlighted trust in HIV clinicians as important sources of knowledge within the changing healthcare system [7, 8]. Given this, we wanted to explore trust in HIV clinicians for a nonurban southern population, as well as trust in other clinicians, insurance companies, and governmental bodies. For attitudes, it has been shown that better ACA knowledge is associated with increased favorability of the ACA [13, 14].

The specific objective of this study was to explore ACA knowledge, HIV-related stigma, trust in various healthcare and governmental bodies, and attitudes toward healthcare and the ACA among PLWH covered by ADAP-funded QHPs in Virginia. This current work adds to the literature by offering more perspectives from PLWH who live in the nonurban US, as they likely have different experiences than those in urban centers. Moreover, the surveys were conducted with individuals, so participants were not influenced by dominant respondents as can happen in focus groups. Exploring knowledge, stigma, trust, and attitudes towards new healthcare policies and their interactions may help guide future interventions in designing health policy or education related to health system delivery changes.

## 2. Methods

### 2.1. Study Enrollment

This prospective study's goal for recruitment was to enroll at least 5% of people who were eligible for ADAP-funded QHPs in two Virginia Department of Health planning regions (Northwest and Southwest). The University of Virginia Institutional Review Board (IRB) for Social and Behavioral Sciences and the Centra Health IRB approved this study. Participants recruited for the study were English-speaking people living with HIV (PLWH) and were eligible for a Virginia ADAP-funded QHP. They were recruited face to face before or after an HIV medical visit in a medical exam room to ensure privacy and confidentiality. Recruitment took place at three Ryan White HIV/AIDS Program (RWHAP) clinics between December 2015–May 2016 and January 2017-February 2017. Participation in the study took an average of 45 minutes, and participants received compensation for their time. The study included a survey and an interview, both of which were administered verbally to minimize any barriers related to low literacy. Findings from the interviews are published elsewhere [9].

### 2.2. Cohort Characteristics

Participants were surveyed with validated measurement tools, when possible. Baseline characteristics collected included demographic, socioeconomic, and HIV-related information. Characteristics included age, self-reported gender, race/ethnicity, financial status (annual income as a percentage of the federal poverty level (FPL)), highest level of education completed, housing stability using methods from Montgomery et al. [15], transportation difficulties, internet access, and mental health including depressive symptoms assessed using the 5-item MHI scale from Berwick et al. [16], problem drinking assessed using the AUDIT-C questionnaire from Bush et al. [17], and a single-question screening test for illicit drug use [18]. Other information related to HIV care that was collected included self-reported current antiretroviral prescription status (yes/no) and self-reported current viral suppression status (virally suppressed, not virally suppressed, and unsure).

### 2.3. Variables

#### 2.3.1. Sources of ACA Knowledge

Participants were provided with a list of possible sources of information about the ACA, which included physician, nurse, clinic case managers, clinic social workers, clinic support staff, other hospital staff, television or magazines, websites, social networking sites, radio, mail, your health insurance company, and friends or family. From this list, they were asked to select their primary source of information and then all sources of information used.

#### 2.3.2. ACA Knowledge

Adapted from a previous study, the following questions were used to assess ACA-related knowledge with the answer options yes, no, and I don't know [19]:

1. Does the Affordable Care Act provide subsidies for people with low incomes to purchase health insurance?
2. Does the Affordable Care Act make it illegal to exclude a person from an insurance plan due to a pre-existing condition?
3. Does the Affordable Care Act eliminate the Ryan White HIV/AIDS Program?
4. Did Virginia decide to move forward with the Affordable Care Act's optional Medicaid expansion?

Answering “I don't know” as an answer choice was considered an incorrect answer. Correct ACA knowledge was defined as getting the first three questions correct, as there was a very low correct response rate about Virginia's Medicaid expansion status.

#### 2.3.3. Stigma

Information regarding HIV-related stigma was collected using the Berger HIV Stigma Scale [20], and the total score was reported. A higher score means that the person is experiencing more stigma.

#### 2.3.4. Trust

Additionally, participants' trust in their main HIV clinician, non-HIV clinician, and health insurance companies was captured by asking their agreement with five statements about each entity [21]. Likert scales were used for these statements, with options including strongly agree, agree, neutral, disagree, and strongly disagree. These were each associated with a numeric score from 5 to 1, and a maximum total score of 25 was possible. Participants' trust in the US federal government and the Virginia state government was also assessed [22]. The questions assessing trust in the US federal government and Virginia state government asked “how often can you trust the governmental body to do what is right,” with answer choices that included always, most of the time, about half the time, some of the time, never, and don't know [22]. The “don't know” answer choice was removed during analysis due to ambiguity.

#### 2.3.5. Attitudes towards the ACA

Participant attitudes were assessed about five topics using a Likert scale as described above: (1) if health insurance helps improve health outcomes, (2) whether the ACA will improve US health outcomes, (3) if they believe they have enough information about the ACA to understand how it will affect their HIV care, (4) if they think the ACA will improve their HIV-related health, and (5) if they believe the ACA will improve their non-HIV-related health. The majority of these questions was adapted from a previous study [19]. The question about having enough information was converted to a binary variable (Strongly Agree/Agree vs. Neutral/Disagree/Strongly Disagree) so that its association with correct ACA knowledge could be studied.

### 2.4. Data Analysis

For statistical testing, all baseline characteristics were collapsed into two or three categories to avoid sparse data bias: age (≤45 vs. >45), gender (cis male vs. noncis male), race/ethnicity (white vs. nonwhite), financial status (≤100% FPL vs. >100% FPL), education level (beyond high school vs. high school and less), housing stability (stable housing, concern for future housing instability, or current unstable housing), transportation difficulties (yes/no), internet access (access to the internet via a computer and a phone, access via only one source point, and no internet access), presence of depression (yes/no, using the MHI-5 scale with 70 points as the cutoff [16]), problematic alcohol use (yes/no, using the AUDIT-C scale with 4 points as the cutoff [17]), and illicit drug use during the past year (yes/no [18]).

Data analysis was performed using R (R Foundation for Statistical Computing, Vienna, Austria) and RStudio (RStudio Inc., Boston, MA). Each question was analyzed with the available data. Any missing data are noted in the results. Descriptive statistics were used to evaluate baseline characteristics, correct ACA knowledge, sources of ACA knowledge, HIV-related stigma, trust in the medical system and government, and attitudes towards the ACA. Mann–Whitney *U* tests or Kruskal–Wallis tests were used to assess for an association between baseline characteristics (age, gender, race/ethnicity, years since HIV diagnosis, financial status, education level, housing stability, transportation difficulties, internet access, depressive symptoms, problem drinking, and illicit drug use) and each of the following: correct ACA knowledge, HIV-related stigma, trust in the medical system and government, and attitudes towards the ACA.

Additional analyses were performed to investigate if there are any associations between correct ACA knowledge and the following data: sources of ACA knowledge, HIV stigma, and trust. A Kruskal–Wallis test was used to assess if any source of knowledge, which was used by at least 5 participants, was associated with a different distribution of correct ACA knowledge questions. Mann–Whitney *U* tests were used to assess the association between correct ACA knowledge and average HIV Stigma Scale overall score and all trust scores. The interaction of participants' perception of having enough information to understand how the ACA will affect their healthcare and performance on the ACA knowledge questions was studied using a Fisher's exact test.

## 3. Results

### 3.1. Participant Characteristics

Characteristics of the participants (*n* = 53) are included in Table 1. We achieved the study enrollment goal of enrolling ≥5% of the PLWH who were eligible for ADAP-funded QHPs in two Virginia Department of Health planning regions (Northwest and Southwest, *n* = 696). The participants all enrolled in an ADAP-funded QHP in the first (2014) or second (2015) year that the option was available. No data were collected on people who did not elect to participate in the survey. The median participant age was 43 years (interquartile range (IQR): 30, 50), and the median time since diagnosis was 10.2 years (IQR: 4.1, 19.7). The majority of participants (66.0%) was male, and just over half (56.6%) were black race/ethnicity. Most participants (69.8%) made less than 133% FPL, and two-thirds completed education equivalent to a high school diploma or less. Nearly 20% of participants reported concerns related to housing stability, about 30% endorsed transportation difficulties, and 17.0% reported having no reliable internet access point. Almost a quarter of participants reported problem drinking or illicit drug use within the past year, while two-thirds endorsed depressive symptoms. More than 90% of participants (92.5%) reported being prescribed ART, and 78.8% of participants reported being virally suppressed.

### 3.2. ACA Knowledge

Almost 80% of participants correctly knew that the ACA provides for low-income subsidies (Table 2). Just over 40% knew that the ACA provides protection for people with pre-existing conditions. Over two-thirds knew that the RWHAP would continue under the ACA. Thirty percent of all participants had correct ACA knowledge. 11% of participants who correctly knew about Virginia's Medicaid expansion status also had correct ACA knowledge. Participants with higher incomes were more likely to demonstrate correct ACA knowledge than those with lower incomes (48% vs. 19%; *p* = 0.03). No other baseline characteristics were associated with correct ACA knowledge.

### 3.3. Sources of ACA Knowledge

**Figure 1:**
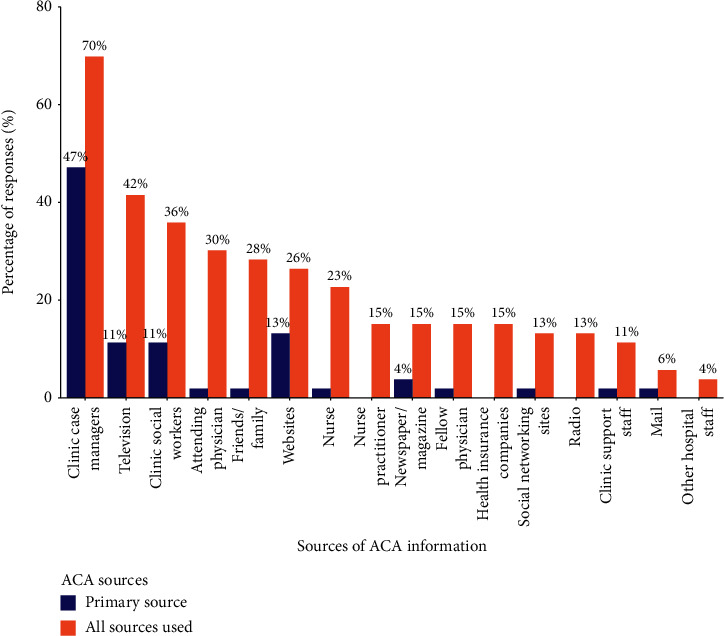
Frequency of participants' sources and main sources of Affordable Care Act knowledge. The labels for bars less than 5% are not shown.

Participants reported that their primary source for obtaining ACA information was clinic case managers (47%) followed by using websites (13%), television (11%), clinic social workers (11%), and newspapers or magazines (4%) (Figure 1). In terms of all sources of information about the ACA, the most common sources were learning from clinic case managers (70%), using television (42%), learning from clinic social workers (36%), learning from an attending physician in charge of their care (30%), and learning from friends or family (28%) (Figure 1). The mean number of reported sources was 3.6 (standard deviation (SD): 2.1; range: 1–10).

Participants who used websites for ACA information were more likely to have correct ACA knowledge compared to those that did not (71% vs. 15%; *p* = 0.001). While not statistically significant, participants who learned ACA information from clinic social workers were more likely to have correct ACA knowledge compared to those that did not (47% vs. 21%; *p* = 0.09). Use of other sources of ACA information (physician, nurse, clinic case managers, clinic support staff, other hospital staff, television or magazines, social networking sites, radio, mail, health insurance companies, and friends or family) was also not associated with correct ACA knowledge.

### 3.4. Stigma

Fifty-one out of 53 participants completed all 40 questions of the Berger HIV Stigma Scale. The overall average stigma score was 104 (SD: 20.0; maximum score: 160; Table 2).

The mean overall stigma scores were higher for participants who were older than 45 years (115.0; SD: 21.2; Table 3) compared to those under 45 years old (97.1; SD: 16.0; *p* = 0.003). They also differed for those who had transportation difficulties (115.0; SD: 17.2) compared to those who had stable transportation (99.4; SD: 19.4; *p* = 0.01). Mean stigma scores were higher for those who did not have internet access (122.0; SD: 16.9) compared to those who had internet access on a computer or a phone (105; SD: 14.8) and those who had access on both a computer and a phone (99.2; SD: 19.5; *p* = 0.01). Lastly, participants with depressive symptoms had higher mean stigma scores (111.0; SD: 19.2) than those who did not have depressive symptoms (90.8; SD: 14.3; *p* = 0.001). Gender, race/ethnicity, financial status, education level, housing stability, problem drinking, and illicit drug use were not associated with differences in overall HIV-related stigma.

Those with correct ACA knowledge had decreased overall stigma scores (93.8; SD: 15.4) compared to those without correct ACA knowledge (108; SD: 20.3; *p* = 0.01).

### 3.5. Trust

The overall average trust score in HIV clinicians was 21.8 (SD: 2.5; max score: 25; Table 2). Overall, the average trust score for general clinicians was 19.5 (SD: 3.6; max score: 25). The overall average score for participant trust in insurance companies was 13.6 (SD: 3.8; max score: 25).

Differences in trust in HIV clinicians were not associated with any baseline characteristics or with correct ACA knowledge. Correct ACA knowledge was associated with lower trust in general clinicians (18.1; SD: 3.8) compared to participants with incorrect ACA knowledge (20.1; SD: 3.3; *p* = 0.01). No other baseline characteristics were associated with differences in trust in general clinicians. Participants who had an education level of high school or less trusted health insurance companies more (14.7; SD: 3.4) than those who had education beyond high school (11.6; SD: 3.9; *p* = 0.008). Other baseline characteristics and correct ACA knowledge were not associated with differences in trust in health insurance companies.

In terms of trust in the federal government, 3.8% said they could “always” trust the federal government, 15.1% said “most of the time,” 22.6% said “about half the time,” 28.3% said “some of the time,” 15.1% said “never,” and 15.1% said “don't know” (Table 2). In terms of trust in the Virginia state government, 5.7% said they could “always” trust the Virginia state government, 17.0% said “most of the time,” 24.5% said “about half the time,” 22.6% said “some of the time,” 15.1% said “never,” and 15.1% said “don't know.”

Differences in trust in the federal government were not associated with any baseline characteristics or with correct ACA knowledge. Participants with depressive symptoms had less trust in the Virginia state government (2.43 points; SD: 1.1) compared to those without depressive symptoms (3.27; SD: 1.2; *p* = 0.02). Other baseline characteristics and correct ACA knowledge were not associated with differences in trust in the Virginia state government.

### 3.6. Attitudes towards the ACA

The mean response for the statement “You believe that having health insurance improves one's healthcare” was 3.9 (SD: 1.0; Table 2). The mean score for the question “Do you think that the Affordable Care Act will improve US health outcomes?” was 3.5 (SD: 0.9). Participants' mean response to “Do you think that the Affordable Care Act will improve your HIV-related health?” was 3.7 (SD: 1.0). The mean response for the question “Do you think that the Affordable Care Act will improve your health?” was 3.4 (SD: 0.9). Over half of participants (53%) agree that they believe that they have enough information about the ACA to understand its impact on their HIV care.

Participants who reported a history of problematic alcohol use were less likely to believe that the ACA would improve their non-HIV-related health (2.9; SD: 1.1) compared with those who did not have problematic alcohol use (3.5; SD: 0.9; *p* = 0.04). Those who reported using an illicit substance in the past year were more likely to say that they did not have enough information about the ACA to understand how it will impact their HIV care (75% vs. 39%; *p* = 0.03). No other baseline characteristics were associated with differences in attitudes towards the ACA. No association was found between a participant having good ACA knowledge and saying they have enough information about the ACA to understand how it will impact their HIV care.

## 4. Discussion

This study highlights that participants had knowledge gaps related to the ACA. Like many PLWH, especially in the south, a significant portion of participants in this study had major barriers to healthcare access including unstable housing, transportation difficulties, a lack of internet access, and high HIV-related stigma scores. There was no association between a participant having correct ACA knowledge and their feeling as though they had enough ACA information to understand how it will affect their HIV care. In light of this finding, HIV clinicians and HIV clinic staff should consider that PLWH may not recognize their own knowledge gaps.

Nearly one-third of the study participants did not know that the RWHAP would be continuing under the ACA, and just over 10% of participants correctly knew that Virginia had not expanded Medicaid at the time of the survey. A 2013-2014 Nebraska study investigating a similar population of PLWH found that only 25% knew about the preservation of the RWHAP, and 63% did not know about whether Nebraska decided to expand Medicaid [10]. This suggests that lack of knowledge about specific aspects of the ACA may be common among PLWH. Our team performed a 2015 study assessing national HIV clinician knowledge of the ACA that showed that a majority of HIV clinicians knew about the preservation of the RWHAP (91%) and their state's Medicaid expansion status (73%). We performed a follow-up study in 2018 that demonstrated HIV clinicians' improved knowledge on these topics as well [23]. This suggests that HIV clinicians can share with PLWH about these topics [19] as well as about the association between ACA Qualified Health Plans and viral suppression [4–6]. From this study, it seems that only one-third of PLWH received any ACA information from their HIV clinician, so this is an area for improvement. Increasing dissemination of this information to PLWH is an important goal for HIV clinicians and HIV clinic staff, such as medical case managers, so that PLWH will have actionable and correct knowledge about the ACA and can advocate for themselves. Excellent skills in system-based medicine have been noted to be an important skill for infectious diseases clinicians [24]. The Accreditation Council for Graduate Medical Education defines system-based medicine as an awareness of and responsiveness to the larger context and system of healthcare, including the social determinants of health, as well as the ability to call effectively on other resources in the system to provide optimal healthcare [25]. Skills in this area may be even more important for HIV clinicians given the barriers that their patients face issues related to social determinants of health that have only been exasperated by COVID-19 [26] and the known impact of social determinants of health on HIV outcomes [27].

Our study population demonstrated high levels of trust in both their HIV clinicians and general clinicians, relative to their trust in health insurance companies. Additionally, for this study population, no baseline characteristic was associated with the difference in trust in HIV clinicians, suggesting that the HIV clinicians are maintaining the trust of PLWH of different ages, genders, race/ethnicity groups, and socioeconomic groups. Previous studies have demonstrated that African Americans may trust their HIV clinicians less than those of other race/ethnicity groups [28]. Trust in clinicians has been shown to be an important factor in care for PLWH, including that it is associated with adherence to antiretroviral therapy [29] and improved retention in HIV care [30]. Trust in clinicians can allow them to become key information brokers related to healthcare, public health, and research [31].

Given the observed trust in the HIV clinician relationship in this population, it seems that there may be an opportunity for education about the ACA to be brokered through HIV clinics by HIV clinicians or case managers [32]. We did not find any association between correct ACA knowledge and learning ACA information from HIV clinicians and HIV clinic staff. However, future strategies to combine trusted and commonly used sources, HIV clinicians and case managers, with websites, which were the only source of ACA knowledge in this study that was associated with correct ACA knowledge, should be explored. While there will be variable health insurance literacy [33], HIV clinics could develop low-cost websites with videos to share accurate and actionable ACA knowledge with PLWH. Videos could be disseminated via a private YouTube channel or a clinic-specific mobile health application. For example, an HIV clinic-based mobile health application utilized its platform to share how the ACA was going to impact HIV care in Virginia [34]. Sharing information electronically reaches PLWH outside of their busy HIV clinic visits, and if it is asynchronous, it could be viewed at a time that is convenient for them. AIDS Education and Training Centers are poised to organize these efforts, as they have a track record in creating changes in clinician practices and changes to the care system [35].

Additionally, given that using websites was associated with correct ACA knowledge, access to the internet is important for PLWH to gain accurate knowledge about healthcare system changes. Internet access is being increasingly recognized as a social determinant of health, and this has been supported by the Federal Communications Commission [36]. Advocating for increased broadband in rural areas and access to smartphones for all PLWH is essential to ensure equitable access to health information [37]. In addition to knowledge benefits, HIV stigma scores were lower with increasing access to the internet.

Additional work needs to be done to understand the interaction between internet access, stigma, and correct knowledge. As mentioned previously, stigma has been called one of the most significant barriers to ending the HIV epidemic [11]. Participants with higher stigma scores were less likely to demonstrate correct ACA knowledge. This raises the question of whether having good knowledge of the healthcare system helps decrease stigma, or if factors that contribute to higher stigma scores are also barriers to accessing knowledge of the healthcare system. Our data revealed similar patterns about what characteristics (increased age, lower incomes, and mental health challenges) are associated with HIV-related stigma as many previous studies [38].

Compared with HIV clinicians and general clinicians, there was lower trust for health insurance companies. This is not surprising given that, before the ACA was passed in 2010, HIV was essentially an uninsurable pre-existing condition in the private marketplace [39]. Due to this issue, having health insurance is relatively new for many PLWH. After the full implementation of the ACA in 2014, the percentage of PLWH with private insurance was estimated to double [1]. It was surprising that the entities that support the RWHAP and ADAP, the federal and state government, did not seem to have much trust from the participants. The RWHAP clinics, ADAP, and ADAP-supported QHPs were generally viewed as beneficial or necessary in the qualitative analysis of this same population's interviews [9] and in the Kaiser Family Foundation's focus group studies [7, 8]. Based on the findings from these participants, it is possible that any goodwill accorded to the governmental bodies for these programs is outweighed by other laws or policies. Looking at trust overall, this study's results suggest that new initiatives or changes to how healthcare is delivered to PLWH may be better accepted if they are communicated from HIV or general clinicians, rather than coming directly from insurance companies or the government. Given the constraints of clinic flow and timing, these messages may need to be electronically delivered, as discussed above.

In terms of looking at specific groups that may need more educational outreach, participants who engaged in illicit drug use reported that they did not feel that they had enough information about the ACA to make informed decisions about their health. When examining ACA attitudes, participants with a history of problem drinking were less likely to believe that the ACA will improve their non-HIV-related healthcare. This could be due to them factoring in their own personal experience or the historically low treatment rates (10%) for people with alcohol use disorder [40]. However, due to the ACA, QHPs must cover Essential Health Benefits which include substance use disorder treatment. There is some leeway at the state level to mandate what exact services must be covered, but this is the first time that any treatment for substance use disorder has to be covered in the US [41].

This study has several limitations including that there was no information collected on those who chose not to participate and the possibility of unmeasured confounding. Moreover, ACA knowledge was measured with only 4 questions. The findings may not be representative of the US given the limited geographic scope. Additionally, all participants were enrolled in ADAP-funded QHPs and received care at RWHAP clinics, which means there was homogeneity in how participants' care was being supported and delivered. The study also has a small sample size. Additional research is needed in larger groups. Participants were recruited from HIV clinic visits, so this population is likely more engaged with the healthcare system and may have a more positive view of the healthcare system than people who are not regularly seeking care. Lastly, as a hypothesis-generating study, we did not use a Bonferroni correction, given that it is a conservative test that protects from type I error, but increases type II errors [42]. Results were presented as they were calculated, and readers should interpret the results in the context of the overall descriptive study.

Since this survey was completed, Virginia has expanded Medicaid [43], so it is possible that people's method or ability to access healthcare, attitudes toward the ACA, or correct ACA knowledge may have changed. Nevertheless, the healthcare system will continue to shift and change. PLWH may not be aware of their knowledge gaps, as systems change. HIV clinicians are a trusted source, HIV case managers are a highly utilized source, and websites are associated with correct ACA knowledge. Combining these three, internet-based videos of HIV clinicians and case managers could help to educate PLWH about the ACA and its impact on HIV care delivery. Lack of internet and stigma pose threats and need to be addressed. Future larger studies should examine how knowledge, stigma, trust, and attitudes may impact the healthcare decisions of PLWH.

## Figures and Tables

**Table 1:** Baseline characteristics.

Cohort characteristics	Total: *n* (%) (*n* = 53)
Age (years)	
Median (IQR)	43 [30, 50]

Gender	
Male	35 (66.0%)
Noncis male	18 (34.0%)

Race	
Black	30 (56.6%)
White	19 (35.8%)
Others	4 (7.5%)

Years since HIV diagnosis	
Median (IQR)	10.3 [4.8, 19.7]

Financial status^1^	
<50% FPL	16 (30.2%)
51–100% FPL	16 (30.2%)
101–133% FPL	5 (9.4%)
134–200% FPL	7 (13.2%)
>201% FPL	9 (17.0%)

Education	
Less than high school	5 (9.4%)
High school or equivalent	30 (56.6%)
Vocational	5 (9.4%)
College degree	11 (20.8%)
More than college degree	2 (3.8%)

Housing stability^1^	
Unstable housing	3 (5.7%)
Stable housing with future concern	7 (13.2%)
Stable housing without future concern	43 (81.1%)

Transportation access	
Difficulty	15 (28.3%)
No difficulty	38 (71.7%)

Internet access	
Neither	9 (17.0%)
Smartphone only	6 (11.3%)
Computer only	2 (3.8%)
Computer and smartphone	36 (67.9%)

Depressive symptoms^2^	
Yes	35 (66.0%)
No	18 (34.0%)

Problem drinking^3^	
Yes	12 (22.6%)
No	41 (77.4%)

Drug use within the past year^4^	
Yes	12 (22.6%)
No	41 (77.4%)

Currently prescribed ART	
Yes	49 (92.5%)
No	4 (7.5%)

Current viral suppression status	
Virally suppressed	41 (78.8%)
Not virally suppressed	6 (11.5%)
Unsure	5 (9.6%)

^1^Housing stability assessed using methods from Montgomery et al. [15]. ^2^Depressive symptoms assessed using the 5-item MHI scale from Berwick et al. [16]. ^3^Problem drinking assessed using the AUDIT-C questionnaire from Bush et al. [17]. ^4^Drug use assessed using a single-question screening test from Smith et al. [18]. Abbreviations: IQR: interquartile range, FPL: federal poverty level, and ART: antiretroviral therapy.

**Table 2:** Respondents' Affordable Care Act (ACA) knowledge, HIV stigma, trust in the healthcare system and government, and ACA attitudes.

ACA knowledge^1^: *n* (%)	Overall *n* = 53
ACA subsidies	
Correct	41 (77%)
Not correct	12 (23%)

Pre-existing conditions	
Correct	23 (43%)
Not correct	30 (57%)

ACA/Ryan White interaction	
Correct	37 (70%)
Not correct	16 (30%)

Virginia Medicaid expansion	
Correct	6 (11%)
Not correct	47 (89%)

Correct ACA knowledge^2^	
Yes	16 (30%)
No	37 (70%)

HIV Berger Stigma Scale^3^: mean (SD)	
Total stigma	104 (20.0)

Trust in clinicians/insurance companies^4^	
Mean (SD)	
Trust in HIV clinicians	21.8 (2.5)
Trust in general clinicians	19.5 (3.6)
Trust in insurance companies	13.6 (3.8)

Trust in governmental bodies^5^: *n* (%)	
How often can you trust the federal government to do what is right?	
Always	2 (3.8%)
Most of the time	8 (15.1%)
About half the time	12 (22.6%)
Some of the time	15 (28.3%)
Never	8 (15.1%)
Don't know	8 (15.1%)

How often can you trust the Virginia state government to do what is right?	
Always	3 (5.7%)
Most of the time	9 (17.0%)
About half the time	13 (24.5%)
Some of the time	12 (22.6%)
Never	8 (15.1%)
Don't know	8 (15.1%)

Attitudes toward the ACA^1^: mean (SD)	
Does insurance improve healthcare?	3.9 (1.0)
Will the ACA improve US health outcomes?	3.5 (0.9)
Will the ACA improve your HIV health outcomes?	3.7 (1.0)
Will the ACA improve your non-HIV health outcomes?	3.4 (0.9)

Do you have enough information on the ACA to understand its impact on your HIV care?	
Agree	28 (53%)
Disagree	25 (47%)

^1^ACA knowledge and attitudes toward the ACA were assessed using questions from McManus et al. [19]. ^2^ Correct ACA knowledge was defined as getting the first three questions correct (ACA subsidies, pre-existing conditions, and ACA/Ryan White interaction) as there was a very low correct response rate about Virginia's Medicaid expansion status. ^3^Stigma was assessed using the Berger HIV Stigma Scale [20]. ^4^Trust in clinicians and insurance companies was assessed using methods from Dugan et al. [21]. ^5^Trust in governmental bodies was assessed using methods from the American National Election Studies [22].

**Table 3:** Stigma score compared to selected baseline characteristics and correct Affordable Care Act knowledge.

	Total stigma score	*p* value
	Mean (SD)	
All participants (*n* = 52)^1^	104 (20.0)	
Age (years)		
≤45 (*n* = 32)	97.1 (16)	0.003
>45 (*n* = 20)	115 (21.2)	

Income		
≤100% FPL (*n* = 32)	108 (20.4)	0.1
>100% FPL (*n* = 20)	97.0 (17.7)	

Stable transportation		
Yes (*n* = 38)	99.4 (19.4)	0.01
No (*n* = 14)	115 (17.2)	

Internet access		
Both (*n* = 35)	99.2 (19.5)	0.01
Computer or phone (*n* = 8)	105 (14.8)	
Neither (*n* = 9)	122 (16.9)	

Depression		
Yes (*n* = 35)	111 (19.2)	0.001
No (*n* = 17)	90.8 (14.3)	

Correct ACA knowledge		
Yes (*n* = 16)	93.8 (15.4)	0.01
No (*n* = 35)	108 (20.3)	

Differences in stigma scores for baseline characteristics were examined with Mann–Whitney *U* tests or a Kruskal–Wallis test (internet access). Differences between stigma scores and correct Affordable Care Act knowledge were evaluated with a Mann–Whitney *U* test. ^1^One participant did not fill out a sufficient number of questions to be included.

## Data Availability

Access to the survey data used for this study is restricted by the University of Virginia Institutional Review Board in order to protect patient's privacy. Data are available from Dr. McManus for researchers who meet the criteria for access to confidential data.
